# P53 and the Ultraviolet Radiation-Induced Skin Response: Finding the Light in the Darkness of Triggered Carcinogenesis

**DOI:** 10.3390/cancers16233978

**Published:** 2024-11-27

**Authors:** Carla Carvalho, Rita Silva, Teresa M. V. D. Pinho e Melo, Alberto Inga, Lucília Saraiva

**Affiliations:** 1 LAQV/REQUIMTE, Laboratόrio de Microbiologia, Departamento de Ciências Biolόgicas, Faculdade de Farmácia, Universidade do Porto, 4050-313 Porto, Portugal; up202010737@up.pt (C.C.); up201904678@up.pt (R.S.); 2 University of Coimbra, Coimbra Chemistry Centre-Institute of Molecular Sciences and Department of Chemistry, 3004-535 Coimbra, Portugal; tmelo@ci.uc.pt; 3 Laboratory of Transcriptional Networks, Department of Cellular, Computational and Integrative Biology, CIBIO, University of Trento, Via Sommarive 9, 38123 Trento, Italy; alberto.inga@unitn.it

**Keywords:** skin carcinogenesis, p53, ultraviolet radiation, chemoprevention

## Abstract

This review delves into the significant cellular and molecular responses triggered by UVR exposure in human skin, emphasizing the pivotal role of mutant p53 (mutp53) in the carcinogenic process elicited by radiation. By underlining the role of a functional p53 in safeguarding skin cells from UVR-induced damage, this work underscores the potential significance of targeting mutp53, aiming to restore its wild-type-like activity (reactivation), as a protective strategy against skin cancer (SC), particularly NMSC. Most importantly, an interesting crosstalk between p53 and its vitamin D receptor (VDR) transcriptional target is also highlighted in the suppression of skin carcinogenesis, which opens the way to promising chemopreventive strategies involving synergistic combinations between mutp53 reactivators and vitamin D. Collectively, this review not only opens new avenues for future research, but also offers promising prospects for the development of novel beneficial approaches in the field of SC.

## Simple Summary

This work examines how ultraviolet radiation (UVR) affects human skin at cellular and molecular levels, particularly focusing on the role of mutant p53 (mutp53) in skin cancer development. It highlights the importance of functional p53 in protecting skin cells from UVR damage, also suggesting that restoring wild-type p53 function could be a valuable strategy against skin cancer, especially non-melanoma skin cancers (NMSC). It further addresses the interaction between p53 and vitamin D receptor (VDR), which may aid in suppressing skin carcinogenesis. This work suggests new chemopreventive strategies that could combine mutp53 reactivators with vitamin D, paving the way for innovative therapeutic approaches in skin cancer prevention.

## 1. Introduction

Skin cancer (SC), including melanoma and non-melanoma skin cancer (NMSC) subtypes, is the fourth most common cancer worldwide. Over the last decades, SC incidence has grown significantly, mainly due to NMSC cases, which are four-fold higher than melanoma [1]. The majority of NMSCs are basal cell carcinomas (BCC), from basal cells of the interfollicular epidermal layer, and cutaneous squamous cell carcinomas (cSCCs), from keratinocytes of the interfollicular epidermal layer and hair follicle stem cells. NMSCs occur predominantly in skin areas exposed to the sun, whereas cutaneous melanomas, which arise from skin-resident melanocytes, are homogeneously distributed throughout the body [2]. Despite having lower mortality rates than melanoma, the disease burden attributable to NMSC will continue to increase in the near future [3]. This fact has been largely attributed to the rising exposure of the population to its main environmental etiological factor, ultraviolet radiation (UVR) [4].

The solar UVR that reaches the earth’s surface is mainly composed of the less energetic UVA rays and 5 to 10% of UVB, depending on different geographic and climatic factors [5]. On the skin, UVA and UVB differ in their depth of penetration through the skin layers. Most UVB radiation can reach the epidermal stratum corneum layer and the upper dermis, leading to dermal changes through epidermis-to-dermis signaling, while long-wave UVA penetrates more deeply into the dermis, reaching the subcutaneous layer of the skin [6]. Some studies report UVA as far less carcinogenic than UVB. Nevertheless, since UVA is more abundant in sunlight and penetrates more deeply, it also plays a role in promoting skin carcinogenesis [7,8]. Although there are some reports of UVC-induced skin mutagenesis, this radiation is absorbed by the stratospheric ozone layer [9]. As such, reported data in the literature are mostly related to UVA and UVB radiations. Despite this, the biological effects of the different types of UV rays are not always distinct, as overlapping cutaneous biological effects are commonly observed [10]. As such, in this review, the generic outcomes of UVR will be considered.

**Figure 1:**
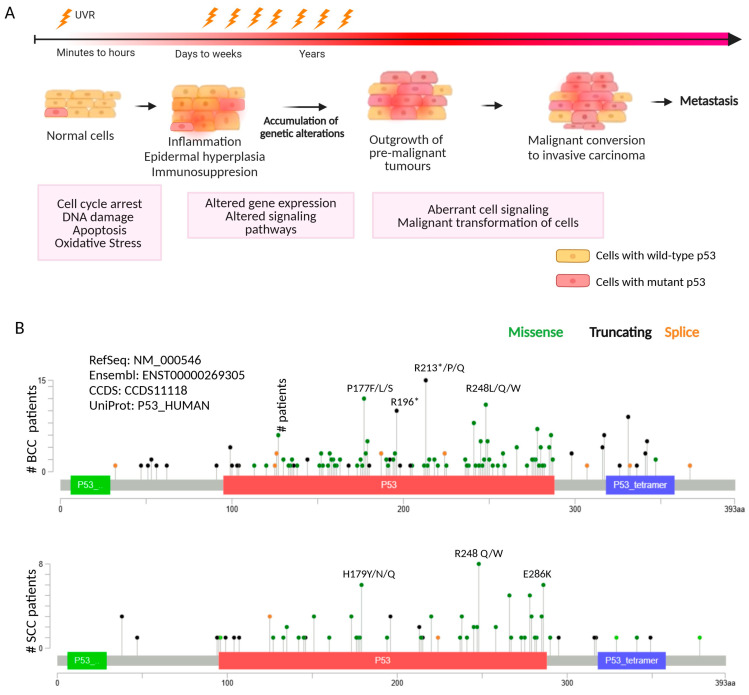
Overall burden of p53 mutations during the time course of ultraviolet (UVR)-induced responses in the skin. (**A**) An acute exposure to UVR (represented by one lightning bolt) promptly generates DNA lesions that are potentially mutagenic and carcinogenic. To survive UVR-induced damage, skin cells activate a variety of signaling pathways that integrate a DNA damage response, modulated by the wild-type p53 (wtp53) tumor suppressor protein (represented by a light pink square), which promotes cell cycle arrest to repair DNA damage or induces apoptosis, according to the severity of the damage. Under UVR-generated ROS, which activate different signaling pathways, wtp53 promotes the transcription of antioxidant enzymes that contribute to the detoxification of ROS. In case of severe UVR exposure, during the following days and weeks, the skin exhibits tissue-level responses such as epidermal hyperplasia, inflammation, and immunosuppression. Repeated UVR exposure of the skin (represented by several lightning bolts) promotes the clonal expansion of the cells and a further increase in the burden of genetic alterations, namely in TP53 (mutant p53 is represented by a dark pink star), where the aberrant cell signaling drives transformation of normal skin cells to premalignant lesions and, eventually, the cells may evolve into invasive and metastatic SC. Non-melanoma skin cancers frequently harbor p53 mutations, leading to the loss of wtp53 functions and the subsequent acquisition of oncogenic properties, such as resistance to apoptosis. Based on [19]. (**B**) Mutational profile of a full sample set of 293 BCC samples [22] and in a combined study with a total of 108 SCC samples [23,24] along the TP53 protein diagrams. The black lollipops represent truncating mutations, the green lollipops represent missense mutation, and the orange lollipops represent splice. Different mutations can occur at the same position; however, the color of the lollipop reflects the mutation frequency at that spot. Hotspot mutations are labeled, including amino acid substitutions or replacements by a stop codon (asterisk [*]). Protein functional domains are represented by colored boxes: the transactivation, DNA-binding, and tetramerization domains are represented by green, red, and blue boxes, respectively. Protein diagrams were generated with cBioPortal tools. SC, skin cancer; UVR, ultraviolet radiation.

Different molecular events have been identified in UVR-exposed skin cells (Figure 1A). In general, acute UVR exposures result in extensive DNA lesions, which trigger DNA damage response pathways. In these cellular responses to UVR, the tumor suppressor p53 is a critical molecular mediator. In fact, p53 accumulation (due to increased half-life of the protein) was detected in UVR-exposed skin cells, where p53 plays an active function as a transcriptional regulator [11]. Low UVR levels trigger a transient p53 activation with subsequent induction of cell cycle arrest, allowing DNA damage repair, while high UVR levels lead to a more pronounced and sustained p53 activation, triggering apoptosis [12]. However, signaling pathways involving phosphoinositide 3-kinase (PI3K), serine/threonine protein kinase AKT, and mammalian (or mechanistic) target of rapamycin (mTOR) (PI3K/AKT/mTOR) can counteract the p53 signaling by activating cell cycle transition, eventually leading to hyper-proliferation and malignant transformation [13]. Alternatively, p53 can activate autophagy or induce premature senescence of the cells to prevent their oncogenic transformation [14]. Therefore, shifting the balance between these signaling pathways can determine the outcome between cell death and survival in irradiated cells [15]. UVR also produces excessive amounts of reactive oxygen species (ROS) that can overwhelm the intracellular antioxidant molecules and activate signaling pathways related to inflammation and carcinogenesis [16]. Additionally, UVR can trigger an immune response that differs according to the wavelength and dose of UVR [17]. Although UVR induces the release of immunostimulatory and proinflammatory cytokines in skin cells, it can also stimulate immunosuppression [17]. In fact, different studies have reported that UVR-induced inflammation stimulates a counteracting immunosuppression involving the expansion of immunosuppressive skin cells [18]. Accordingly, cumulative UVR exposures are associated with sustained immunosuppression accumulation of genetic modifications, and aberrant signaling pathways, promoting skin carcinogenesis [19].

In cumulative UVR exposures, the consequent increased mutational burden includes an accumulation of *TP53* mutations, which plays a critical role in the development of skin carcinogenesis. In fact, the presence of mutant p53 (mutp53) has been reported in around 50% of BCC and over 90% of SCC [20]. Particularly, mutp53 is found in pre-malignant lesions of SCC, the actinic keratosis (AK), supporting its early involvement in SCC [21]. Figure 1B depicts the mutational profiles interpreted with protein annotation of p53 in BCC [22] and SCC samples from the latest cancer studies [23,24].

This review delves into the significant cellular and molecular responses induced by UVR exposure in human skin, emphasizing the pivotal role of mutp53 in skin damage triggered by radiation. The potential use of mutp53 reactivators to counteract skin carcinogenesis, alone or in combination with other chemopreventive agents, is also addressed.

## 2. Skin Damage Induced by UVR Exposure: The Protective Role of p53

### 2.1. UVR-Induced DNA Damage

The photochemical differences between UVA and UVB radiation activate different cellular responses [25]. Regarding DNA damage, UVB is primarily a nucleotide damaging agent since UVB chromophores are strongly absorbed by the aromatic heterocyclic bases, leading to covalent damage at pyrimidine bases. As a result, cyclobutane pyrimidine dimers (CPDs) and pyrimidine (6-4) pyrimidone photoproducts (6-4PPs) are formed. If unrepaired, these errors can strongly block DNA and RNA polymerases, causing mutagenesis or cell death [26]. UVA mainly produces ROS through interaction with endogenous photosensitizers that can damage DNA, proteins, and membranes via oxidative reactions. The products 8-oxoguanine (8-oxoG) and 8-oxo-2′-deoxyguanosine (8-oxodG), guanine and deoxyguanosine oxidation products, respectively, are the biomarkers for UVA-induced oxidative DNA damage [27]. However, there is some controversy regarding the effects specifically attributed to each UVR. As such, as previously mentioned, the generic effect of UVR will be addressed.

**Figure 2:**
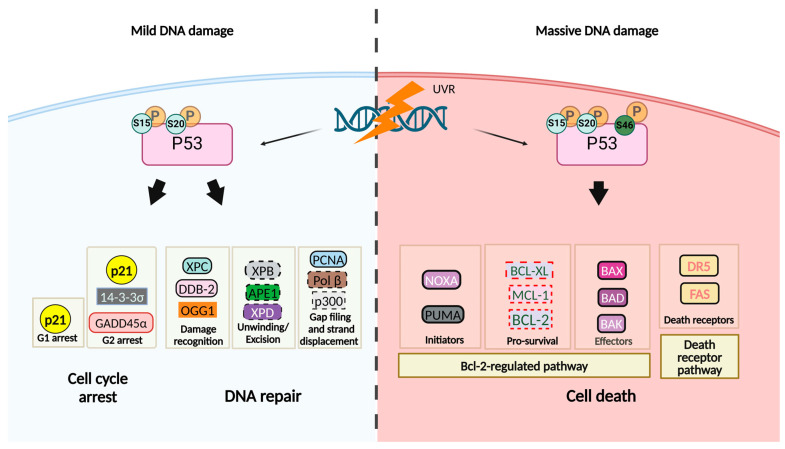
p53 differentially regulates cell fate decisions in response to mild and severe UVR-induced DNA damage. Upon mild UVR-induced DNA damage, p53 is phosphorylated at Ser15 and Ser20 residues by ATM and CHK2, disrupting the interaction with MDM2 and thus promoting p53 stabilization, and subsequent transactivation of p53 target genes. Activated p53 transcriptionally regulates *CDKN1A* (encoding p21) that promotes G1 arrest. p53 also promotes G2 arrest by regulating the transcription of GADD45α and 14-3-3σ, besides p21. During cell cycle arrest, p53 promotes DNA repair through non-transcriptional- (dashed line) and transcription-related proteins (solid line). In response to severe DNA damage, p53 is further phosphorylated at Ser46 by p53 Ser46 kinases, resulting in preferential transactivation of cell death-stimulating p53 target genes. Cell death regulators encoded by direct p53 target genes are outlined in a solid black box (black represents induction); cell death regulators indirectly induced are outlined in a dashed black box; cell death regulators indirectly repressed are outlined in a dashed red box (red represents inhibition). Based on [30]. APE1, apurinic/apyrimidinic Endonuclease 1; ATM, ataxia telangiectasia mutated; BAD, Bcl-2-antagonist of cell death; BAK, Bcl-2 Antagonist/Killer; BAX, Bcl-2-associated X protein; BCL-2 B-cell lymphoma 2; BCL-XL, B-cell lymphoma-extra-large; CDKN1A, Cyclin Dependent Kinase Inhibitor 1A; CHK2, checkpoint kinase 2; DDB2, damaged DNA binding protein 2; DR5, Death Receptor 5; Gadd45α, growth-arrest and DNA damage-inducible; MCL-1, Myeloid Cell Leukemia 1; MDM-2, mouse double minute 2; OGG1, 8-oxoguanine DNA glycosylase 1; PCNA, proliferating cell nuclear antigen; polβ, DNA polymerase β; PUMA, p53-upregulated modulator of apoptosis; ROS, reactive oxygen species; UVR, ultraviolet radiation; XPB, xeroderma pigmentosum group B protein; XPC, xeroderma pigmentosum group C protein; XPD, xeroderma pigmentosum group D protein.

Upon UVR-induced DNA damage, different pathways lead to the dissociation of the p53/mouse double minute 2 (MDM2) inhibitory complex [28] and two major kinases, ataxia telangiectasia mutated (ATM) and ataxia telangiectasia and Rad3-related (ATR), mediate p53 phosphorylation, preventing its degradation [29]. Some of the major cellular responses to UVR, involving p53 activation, are depicted in Figure 2. Upon mild DNA damage, p53 is phosphorylated at serine (Ser)15 and Ser20, disrupting its degradation and thus promoting p53 stabilization and subsequent transactivation of its target genes [30]. ATM can directly phosphorylate p53 at Ser15 and mediate p53 Ser20 phosphorylation via activation of its direct substrate checkpoint kinase 2, CHK2 [29]. Therefore, UVR exposure results in a p53-driven cell cycle arrest by transactivating genes involved in G1 and G2 phases. When UVR-induced DNA damage occurs before entry into the S phase, p53 halts the cell cycle at the G1 phase in part by transcriptionally inducing cyclin-dependent kinase inhibitor (*CDKN1A)*, also known as p21 (WAF1/CIP1), a potent inhibitor of several cyclin-dependent kinase complexes. p21 may also block the elongation step of DNA replication during the S-phase of the cell cycle, or it may inhibit the complexes of cyclin-dependent kinases with both cyclin A and cyclin B, promoting G2 arrest [31]. Gadd45α (growth-arrest and DNA damage-inducible) and 14-3-3σ are also p53 effectors that participate in the control of G2/M transition [32].

During cell cycle arrest, DNA repair mechanisms such as nucleotide excision repair (NER) and base excision repair (BER) are activated, in which p53 plays transcription-dependent and -independent functions [31]. p53 mediates the transcription of genes encoding damaged DNA binding protein 2 (*DDB2/p48*) and xeroderma pigmentosum group C (*XPC*) proteins, involved in the global genome NER that repairs dipyrimidine photoproducts [31]. During the repair process, DDB2 associates with DDB1 to form the UV-DDB heterodimer, which in turn binds to 6-4PPs and CPD lesions, promoting the recruitment of XPC and other proteins, during the early stages of the NER mechanism. Global genome and transcription-coupled NER converge on a common repair pathway with a local DNA relaxation promoted by the multimeric protein, the transcription factor IIH (TFIIH), via the helicase activity of its xeroderma pigmentosum group B (XPB) and D (XPD) subunits, respectively [33]. The p53 transcription-independent functions include the modulation of the helicase activities of XPB and XPD [34] and of chromatin accessibility via p53-dependent recruitment of the p300 histone acetylase to damage sites, where it acetylates histone H3 subunit [35]. Oxidized bases are repaired by the BER mechanism, in which *OGG1* (encoding 8-oxoguanine glycosylase) plays a role in the recognition and excision of 8-oxoG bases [36]. Evidence that *OGG1* is a transcriptional target of p53 is not entirely consistent across all studies. In fact, although some studies have suggested that p53 can upregulate *OGG1* expression [36], there are also studies suggesting an overexpression of *OGG1* through a p53 transactivation-independent manner [37]. p53 stimulates BER also by interacting with apurinic/apyrimidinic Endonuclease 1 (APE1)/Redox Factor 1 (REF1) [38] and DNA polymerase β [33]. Proliferating cell nuclear antigen (*PCNA*), also a p53 transcriptional target, is a major coordinator of faithful and processive replication.

In response to severe DNA damage, p53 is additionally phosphorylated at Ser46 by several p53 Ser46 kinases, including ATM, protein kinase C delta (PKC*δ*), Homeodomain-Interacting Protein Kinase 2 (HIPK2), mammalian dual-specificity tyrosine (Y) phosphorylation-regulated kinase 2 (DYRK2), and MAP kinase p38*α* [30]. Although some data have indicated UVR-induced p53 phosphorylation in other residues, namely Ser6 and Ser392 [39,40], Ser46 is particularly significant for the onset of apoptosis [30]. The additional p53 phosphorylation at Ser46 results in preferential transactivation of cell death-stimulating p53 target genes and p53-dependent induction of mitochondrial outer-membrane permeabilization, involving anti- and pro-apoptotic members of the B-cell lymphoma 2 (BCL-2) family. In fact, UVR-activated p53 promotes apoptosis through transcriptional activation of pro-apoptotic genes, such as p53-upregulated modulator of apoptosis (*PUMA)* [41] and phorbol-12-myristate-13-acetate-induced protein 1 (*PMAIP1*) (encoding NOXA protein) [41], which bind and inhibit the pro-survival BCL-2 proteins, such as BCL-2, BCL-XL, and Myeloid Cell Leukemia 1 (MCL-1) [42], thereby unleashing the pro-apoptotic effectors BCL-2-associated X protein (BAX), BCL-2 Antagonist/Killer (BAK), and Bcl-2-antagonist of cell death (BAD). Additionally, p53 can activate apoptosis-related genes encoding death receptors like Fas (also known as APO1) and KILLER/DR5 (Death Receptor 5) [12,43]. More recently, a critical role of IκB kinase alpha (IKKα) in mediating p53 activation was reported in human keratinocyte and mouse embryonic fibroblasts upon UVB exposure, to determine cell fate [44]. The authors showed that IKKα triggered p53 phosphorylation and acetylation, upregulating the expression of its pro-apoptotic target genes [44].

Although p53 induces apoptosis in response to severe UVR-induced damage, UVR also activates AKT pathways that can counteract p53-mediated apoptosis through AKT-dependent phosphorylation of MDM2, resulting in increased MDM2 stability and malignant transformation of skin cells. In turn, p53-induced apoptotic processes can commit AKT cleavage by executioner caspases [45]. This crucial role of p53 in the elimination of damaged cells is underlined by the clinical evidence that UVR-induced p53 mutations are highly frequent in human SCC and BCC [46], with enrichment for C > T and CC > TT transitions at dipyrimidine sites. Indeed, chronic UVR exposure can promote the clonal expansion of cells carrying the mutated *TP53* gene, fostering skin carcinogenesis [12].

### 2.2. UVR-Induced Oxidative Stress

UVR induces ROS production by changing the peroxidatic activity of the enzyme catalase and upregulating nitric oxide synthase (NOS) synthesis [47]. To strengthen the repair and/or elimination of damaged molecules or the induction of cell death, the radiation-induced ROS, the oxidized products, and the DNA lesions activate different signaling pathways. Against ROS-mediated damage, skin cells exhibit an antioxidant defense that includes nonenzymatic and enzymatic systems and the stabilization of the nuclear factor-E2-related factor (Nrf2) [48]. Nrf2 induces the transcription of antioxidant genes by binding to the antioxidant response element (ARE) in their promoter regions. Nevertheless, UVR-induced ROS can overwhelm the endogenous antioxidant capacity of skin cells, promoting oxidative stress that results in lipid peroxidation, protein oxidation, and DNA damage [48]. Several stress-sensitive kinases are also activated by UVR-induced ROS, playing a pivotal role in transducing the oxidative stress signals into cellular responses, including the expression of proinflammatory cytokines, and consequent promotion of skin inflammation.

UVR itself and induced ROS activate the mitogen-activated protein kinase (MAPK) pathway with final effectors, including c-JUN NH2 terminal kinases, extracellular signal-regulated kinases, ERKs, and p38 kinases that regulate the activity of downstream transcription factors [16]. Extracellular signal-regulated kinases and c-Jun N-terminal Kinase (JNK) promote the phosphorylation and activation of the activating protein-1 transcription factor (AP-1) components, which are primarily proteins from the Fos and Jun families. Once phosphorylated, c-Jun and c-Fos can dimerize to form the AP-1 transcription factor, which translocates to the nucleus, where it binds to specific DNA sequences known as AP-1 binding sites. Activation of p38 and inhibitory kappa kinases (IKK) are required for the transcriptional activation of nuclear factor kappa light chain enhancer of activated B (NF-kB). Both AP-1 and NF-kB are important in regulating a diverse array of genes involved in inflammatory signaling [16].

**Figure 3:**
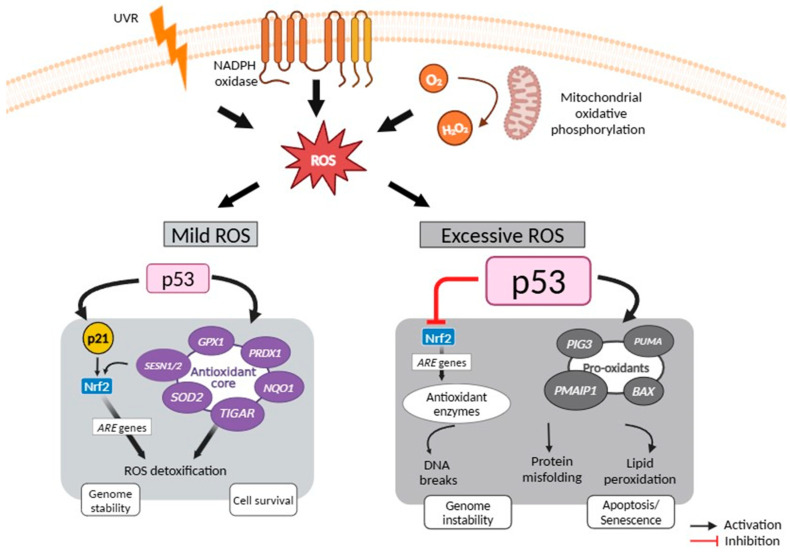
UVR-induced ROS response associated with p53 signaling. At moderately low levels of ROS, p53 exhibits an antioxidant activity mainly by inducing *p21* and *SESN1/2*, which stabilize the antioxidant regulator Nrf2 that leads to transactivation of *ARE* genes, and by inducing targets such as *SOD2*, *TIGAR*, *GPx1*, *PRDX1*, and *NQO1*, ultimately leading to a reduction of oxidative stress. This antioxidant activity protects cells from oxidative stress-induced DNA damage and mutations. At high ROS levels, for example when extensively increased by radiation, p53 can be additionally activated by MAPKs signaling, which is responsible for the upregulation of pro-oxidant genes, including *PIG3*, *BAX*, *PUMA*, and *PMAIP1*, to further induce ROS, which in turn activate p53. Increased p53 levels suppress the Nrf2-mediated cell survival pathway. This pro-oxidant activity leads to apoptosis or senescence to prevent the propagation of mutation-bearing cells. Based on [58]. ARE, antioxidant response element; BAX, Bcl-2-associated X protein; GPx1, glutathione peroxidase; MAPK, mitogen-activated protein kinase; NQO1, NAD(P)H Quinone Dehydrogenase; Nrf2, nuclear factor-E2-related factor; PIG3, tumor protein p53 inducible protein 3; PMAIP1, phorbol-12-myristate-13-acetate-induced protein 1; PRDX1, peroxiredoxin; PUMA, p53-upregulated modulator of apoptosis; ROS, reactive oxygen species; SESN1/2, sestrin 1/2; SOD2, superoxide dismutase 2; TIGAR, TP53-induced glycolysis and apoptosis regulator; TP53INP, tumor protein p53-induced nuclear protein 1; UVR, ultraviolet radiation.

Under low intracellular ROS levels, p53 is activated by ATM and checkpoint kinase 2, CHK2, promoting the transcription of antioxidant enzymes that contribute to the detoxification, and upregulating the levels of reducing molecules, such as nicotinamide adenine dinucleotide phosphate (NADPH) and glutathione. Some examples of p53-activated ROS-detoxifying molecules reported in an UVR context are depicted in Figure 3, including the enzymes manganese superoxide dismutase 2, SOD2 [49], glutathione peroxidase 1, GPx1 [50], peroxiredoxin, PRDX1 [51], and NAD(P)H quinone dehydrogenase 1 (NQO1) [52]. In addition, the p53 transcriptional targets sestrin *SESN1* and *SESN2* interact with Nrf2 antagonist Kelch Like ECH Associated Protein 1 (Keap1), promoting its degradation [53]. The p53 target p21 competes with Keap1 for Nrf2 binding, resulting ultimately in a stable and activated Nrf2 that upregulates the expression of several genes through binding to AREs [54]. Although not reported in a UVR context, the p53 transcriptional target *TP53-induced glycolysis and apoptosis regulator* (*TIGAR*) [55] and *tumor protein p53-induced nuclear protein 1* (*TP53INP1*) [56] also comprise part of the antioxidant p53 core transcriptional program. In addition, the tumor protein p53-induced nuclear protein 1 (TP53INP1) facilitates p53 transcriptional activity directly interacting with p53 and different kinases, also maintaining mitochondrial integrity [57].

High levels of ROS or prolonged stress upregulate p53 and provoke a pro-oxidant response to further increase ROS, which subsequently elicit p53-dependent apoptotic processes to eliminate damaged cells [59]. In addition to directly activating p53, the excessive UVR-induced ROS activate JNK and p38 MAPK, which mediate p53 activation with upregulation of target genes exhibiting a pro-oxidant role such as *BAX*, the tumor protein p53 inducible protein 3 (*TP53I3* or *PIG3*), *PUMA*, and *PMAIP1* (encoding NOXA) [60]. *BAX*, *PUMA*, and *NOXA* contribute indirectly to oxidative stress by promoting mitochondrial dysfunction, while *PIG3* exhibits an oxidoreductase activity, being considered as a direct pro-oxidant [61]. p53 has also been involved in the suppression of antioxidants associated with Nrf2 by blocking the binding of Nrf-2 to the ARE sequences present in their genes [62]. Of note, excessive UVR-induced ROS production, usually associated with cumulative UVR exposures, can also promote senescence, which will be further discussed. Figure 3 highlights the multifaceted activity of p53 that contributes to cell fate decision-making in response to low and high levels of UVR-induced ROS.

### 2.3. UVR-Induced Inflammation

UVR-induced inflammation is strictly associated with the pathogenesis of many skin diseases, including NMSC [63]. Indeed, both acute and chronic UVR skin exposure promote the release of inflammatory mediators in the serum and epidermis and the infiltration of immune cells. As mentioned above, acute UVR increases NADPH oxidase and ROS production. This increase also elicits an inflammatory response in the cutaneous microvasculature, leading to skin erythema (sunburn), production of inflammatory mediators, alteration of vascular responses, and inflammatory cell infiltrates [64]. Chronic UVR promotes a sustained inflammatory environment that compromises cell structure and function, and cutaneous and systemic immunity, including changes in the production of cytokines by skin cells [19], contributing to accelerated skin carcinogenesis. As previously referred, inflammatory pathways can also be activated by UVR-induced ROS or indirectly by DNA or mitochondrial damage, where both keratinocytes and fibroblasts contribute to the inflammatory condition and extracellular matrix degradation. However, keratinocytes are considered as the major source of cytokines production, while fibroblasts mainly secrete matrix metalloproteinases (MMPs) [65]. It was shown that exposure of epidermal keratinocytes to UVA or UVB results in the activation of the major inflammatory mediator NF-κB, inducing pro-inflammatory cytokines [66]. In fact, the p65-dependent NF-κB signaling has been implicated in the promotion of a pro-inflammatory environment in SCC [67].

**Figure 4:**
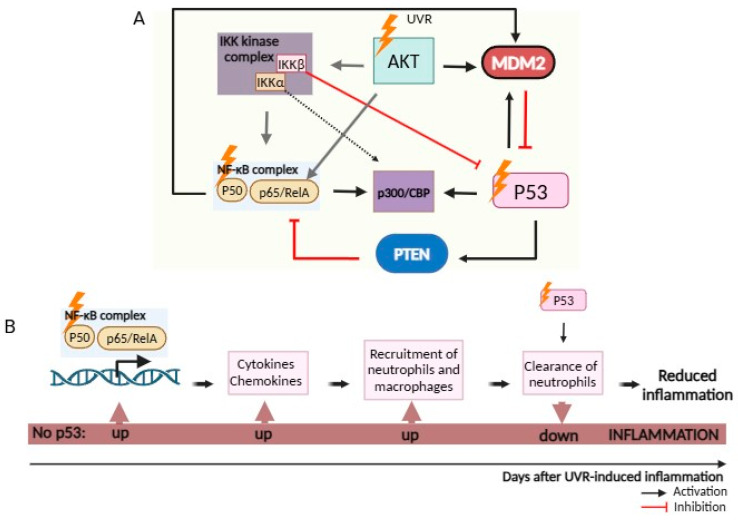
Regulation of UVR-induced skin inflammation by p53. Upon UVR, the crosstalk between wtp53 and NF-κB is mostly mutually inhibitory, although cooperative functional interactions have been also described, depending on UVR-induced damage. (**A**) UVR-activated AKT can activate both IKKα and IKKβ, and phosphorylate the p65 subunit of NF-κB, enhancing its transcriptional activity. To counteract p53 functions, and since there is competition for the limited pool of the coactivator CBP/p300, IKKα mediates phosphorylation of p300/CBP, rendering it a preferential partner of NF-κB, while IKKβ introduces post-translational modifications of p53, compromising its stability. AKT-mediated MDM2 phosphorylation and NF-κB upregulation of *MDM2* also result in p53 inhibition. Instead, p53 inhibits the transactivation activity of the p65 subunit of NF-κB through its target gene *PTEN*. (**B**) UVR-activated NF-κB induces the expression of several pro-inflammatory genes, including those encoding cytokines and chemokines that, in turn, promote neutrophils and macrophage recruitment. In this regard, p53 acts as a general inhibitor of NF-κB-dependent transcription and as a positive regulator of neutrophil clearance by macrophages. Hence, loss of p53 results in over-reaction to pro-inflammatory stimuli. Based on [70]. AKT, serine/threonine protein kinase; CBP, CREB binding protein; IKKα, IκB kinase alpha; IKKβ, IκB kinase beta; MDM-2, mouse double minute 2; NF-κB, nuclear factor kappa light chain enhancer of activated B; PTEN, phosphatase and tensin homolog; UVR, ultraviolet radiation.

NF-κB and p53 antagonistically regulate each other through different mechanisms, whereby NF-κB interferes with p53 protective functions, favoring an inflammatory condition and promoting cellular transformation [68]. Figure 4 depicts the ambivalent relationship between the two transcription factors in UVR-induced inflammation, particularly the molecular mechanisms responsible for reciprocal negative regulation of p53 and NF-κB. In UVR-induced inflammatory processes, NF-κB promotes the secretion of bioactive chemokines and cytokines, such as interleukin 6 (IL-6) and tumor necrosis factor alpha (TNF-α) that stimulate the immune response, promoting the infiltration of neutrophils, which are producers of ROS. To counteract this, p53 acts as a positive regulator of neutrophil clearance by macrophages [69]. In fact, the ectopic expression of p53 was shown to inhibit transcription from NF-kB-dependent promoters and to promote neutrophil clearance by macrophages [70]. However, some studies have already demonstrated that p53 and NF-κB can co-regulate pro-inflammatory genes, such as *IL-6* and *C-X-C Motif Chemokine Ligand 1* (*CXCL1*), in human macrophages, to drive the induction of pro-inflammatory cytokines [71]. Additionally, it was reported that transcriptional activation of the inflammatory mediator cyclooxygenase-2 (COX-2) by p53 requires both NF-κB p50 and p65 proteins, and when NF-κB was inhibited, it significantly reduced COX-2 activation by p53 [72].

In fact, there are many lines of crosstalk between p53 and NF-κB pathways. NF-κB can repress p53 activity through upregulation of the p53 inhibitor *MDM2* [73] Alternatively, the IκB kinase β (IKKβ), a major player in the canonical NF-κB pathway, can induce post-translational modifications in the p53 protein, altering its stability [74] (Figure 4A). On the other hand, UVR-activated p53 can also inhibit NF-κB, namely by blocking its cytoplasmic-to-nuclear translocation [75]. Moreover, the p53 target gene phosphatase and tensin homolog (*PTEN*) was shown to block NF-κB transcription by interfering with the transactivational activity of the p65 subunit [76]. Additionally, since both transcription factors compete for common cofactors, the ability to induce rapid transcriptional responses is highly dependent on inducible changes in chromatin structure. Indeed, both p53 and NF-κB have been shown to repress the activity of each other by competing for limited pools of the transcriptional coactivator proteins p300 and the closely related cAMP response element-binding protein (CREB) binding protein (CBP), which is a transcriptional coactivator that interacts with CREB, being crucial components in the initiation of signal-induced transcription. IKKα mediates phosphorylation of the coactivator p300/CBP, rendering it a preferential partner of NF-κB instead of p53, inhibiting p53 activity [77]. Additionally, UVR-activated AKT can mediate MDM2 phosphorylation to inhibit p53 stabilization [78] and stimulate IKKs, which leads to the phosphorylation and degradation of IκB proteins, thereby activating NF-κB. Moreover, AKT can directly phosphorylate the p65 subunit of NF-κB, enhancing its transcriptional activity [79]. Despite the huge amount of reported data, the mechanisms underlying NF-κB inhibition by p53 remain unclear.

### 2.4. Alternative Processes in UVR Exposure Skin Cells: Autophagy and Senescence

To maintain skin integrity, UVR-activated p53 and PI3K/AKT/mTOR signaling pathways reciprocally interact with each other, emerging as potential life/death regulators of irradiated skin cells. Additionally, in response to UVR, activated p53 can regulate the expression of autophagy modulators and factors mediating inhibition of AKT/mTOR that, ultimately, promote the activation of the autophagy-initiating complex. p53 upregulates the damage-regulated autophagy modulator 1 (*DRAM1*), which codes for a lysosomal protein that is required for the induction of autophagy [80]. p53 also promotes autophagy by upregulating negative regulators of the AKT/mTOR pathway, such as PTEN and sestrins [81]. Furthermore, inhibition of the anti-autophagic mTORC1 can be enhanced by p53-mediated transactivation of tuberous sclerosis complex (TSC)2 that, together with TSC1, blocks mTORC1, enabling the autophagy process [80]. This inhibition of AKT/mTOR, in combination with transactivation of damage-regulated autophagy modulators, guides the p53-mediated elimination of damaged cellular components by autophagic clearance.

p53 has also been described as a key player in the induction of senescence since, in concert with PI3K/AKT/mTOR signaling, it can drive the cells to an irreversible cell cycle arrest that counteracts oncogenic transformation [82]. This response is dependent on different p53 levels, kinetics, alternative splicing of mRNA, post-transcriptional modifications, recruitment of different co-factors, and transcriptional activation of different sets of p53 target genes. Under UVR conditions, these factors are responsible for activating p53, inducing specific patterns of genetic expression that can promote senescence [15]. Several studies have demonstrated that p53 is involved in the earliest stages of senescence via p21/CIP1 activation, revealing its role in the induction of an early phase and still reversible senescence by inhibiting different apoptotic agents to promote senescence [83]. Additionally, UVR is reported to induce the expression of MMPs, which play a role in photocarcinogenesis by promoting tumor invasion and metastasis, including MMP-1 [84], MMP-2 [85], MMP-3 [86], and MMP-9 [85,86]. Different studies correlate the regulation of MMPs by p53 in a UVR context. Namely, it has been shown that p53 downregulates MMP1, a collagenase that degrades collagen, mainly by disrupting the communication between AP-1 and the basal transcriptional complex [87]. There are also studies reporting a p53-dependent inhibition of the gelatinase MMP-9 [88] and collagenase MMP-13 [89] expression. p53 was also demonstrated to potently inhibit MMP-13 and MMP-1 expression, promoting a decreased SCC cell invasion independently of the proapoptotic effect of p53 [90].

## 3. p53 Activation to Counteract UVR-Induced Skin Carcinogenesis

Considering that NMSC risk is strongly associated with UVR, effective preventive strategies should be undertaken to avoid the development of photocarcinogenesis. Despite the common sun protection recommendations, particularly the use of sunscreen, the growing incidence of NMSC motivates the search for complementary protective approaches. Additionally, the associated accumulation of UVR-induced mutp53 in early phases of skin carcinogenesis in NMSC suggests that preventing its formation could be a promising strategy for SC prevention. In fact, Benjamin et al. [11] demonstrated that pre-treatment of mice grafted with human skin samples with sunscreen before UVR exposure promoted the reduction of UVR-induced mutp53 [11]. In addition to sunscreen, different natural and synthetic compounds have been reported to counteract UVR-induced deleterious skin effects, mainly through activation of p53 or of its downstream targets [91].

Disruption of p53 activity by *TP53* mutation, particularly missense mutations, allows cells to bypass repair mechanisms or apoptosis, promoting the clonal expansion of mutated cells and further development of carcinogenesis [92]. Certain mutations cause loss of function of p53, whereas others inhibit p53 function through a mutp53 dominant-negative effect over wild-type p53 proteins [93]. In addition, some missense mutations confer a gain of function activity, namely by altering the ability of mutp53 to bind canonical p53 DNA binding regions, with subsequent regulation of alternative genes [94] and inhibitory interactions with other transcription factors such as p63 and p73 [95]. Sequencing analyses of human cSCCs have revealed numerous point mutations in *TP53*, primarily caused by UVB-induced C > T or CC > TT base substitutions, establishing a link between UVR exposure, DNA damage, and skin carcinogenesis. Indeed, *TP53* point mutations have been identified in approximately 60% of actinic keratoses and 50–90% of cSCCs [96], mainly occurring in DNA-binding domain (DBD) clustering at several hotspot amino-acid residues [23,24]. Particularly, mutp53 R248W is commonly detected in cSCCs, exhibiting oncogenic gain of function properties that promote tumorigenesis [25,26]. Also, mutp53 R175H has been found in head and neck SCCs (HNSCCs) [97] and cutaneous SCCs [98], resulting in impaired zinc coordination that compromises p53 folding and transcriptional activity [97]. Although the exact role of mutp53 in the development of BCC remains not fully elucidated [99], genomic analysis of BCC samples already identified new *TP53* mutations functioning as drivers for skin carcinogenesis [22] (Figure 1B). More recently, *TP53* missense mutations were identified in BCC samples, including hotspot positions p.H179Y, p.S241F, p.G245N, and p.R280K, clustering in p53 DBD [100]. Particularly, R196* was the most prevalent *TP53* nonsense mutation in SC, which results in p53 transcript degradation via the nonsense-mediated decay pathway [22,100,101].

For long, extracts and isolated compounds from diverse plants have been studied for their protective properties against UVR-induced SC [102]. These substances have active principles with antioxidant, anti-inflammatory, and immunomodulatory properties, which aid in controlling dermal extracellular matrix remodeling. Some of these plant-derived compounds exhibit their activities through the regulation of p53 [103]. Particularly, different reports have demonstrated the protective effect of resveratrol in SC, reporting an increased p53 expression [104], as well as anti-inflammatory [105] and anti-proliferative [106] effects. Pomegranate extract has been reported to reduce UVR-induced mutp53 levels [107], also increasing the expression levels of p53 and its transcriptional target p21 [108]. Pomegranate reduces UVR-induced DNA damage [108], promotes apoptosis by transcriptional regulation of *BAX*, *BAD*, and *BCLXL* [109], and reduces UVR-induced oxidative stress and inflammatory effects in mouse models [107]. Silibinin has also been reported for its photoprotective properties [110], increasing p53 levels in response to UVR [111], promoting DNA damage repair [110], and exhibiting immunomodulatory [112], anti-inflammatory [113], and anti-proliferative effects [111]. A protective activity of nicotinamide has also been linked to the regulation of p53 and sirtuins in vitro [114] and in NMSC cases under clinical trials [115]. Protective effects were also reported in NMSC for compounds such as curcumin [116], caffeine [117], glycyrrhizic acid [118], and the β-blocker carvedilol [119], by targeting p53-mediated signaling pathways.

The crucial role of p53 in skin carcinogenesis has been the basis for the development of distinct p53-based therapeutics for NMSCs. In particular, different clinical trials (NCT00041613, NCT00041626) are underway using adenoviral p53 gene therapy in patients with recurrent SCC [120]. Additionally, p53 vaccines, which incorporate p53 peptides, have been tested in clinical trials (NCT00404339) with patients with HNSCC [121].

The high burden of p53 mutations in NMSC makes mutp53 an appealing target in SC prevention. In fact, cumulative data have corroborated the protective activity of several compounds known to restore the wild-type-like function to mutp53 (called reactivators). In particular, it was demonstrated that phenethyl isothiocyanate exhibited antitumor effect in esophageal SCC cells by restoring the transcriptional activity to p53R248Q [122]. Additionally, some known mutp53 reactivators, such as CP-31398 [123] and SLMP53-2 [124] have shown evident protective effects against UVR-induced skin carcinogenesis. CP-31398 binds to the p53 core domain, restoring its DNA-binding ability and promoting p53-dependent cell cycle arrest and apoptosis in UVB-irradiated p53^+/+^ but not in p53^−/−^ skin [123]. SLMP53-2 was able to reactivate mutp53, enhancing DNA repair, suppressing inflammation, and promoting differentiation, in UVB-irradiated keratinocytes and mice models [124]. More recently, the reactivation of mutp53 by p53 conformation activating peptide-250 (pCAP-250) was demonstrated in primary dermal fibroblasts from Li-Fraumeni syndrome patients carrying the mutp53 R248Q [125]. Long-term treatment with pCAP-250 prevented mutp53 accumulation, reduced DNA damage, and restored genomic stability. This interesting work further reinforced the potential application of mutp53 reactivators as protective agents to delay early onset cancer in carriers of mutp53, namely induced by UVR.

It is worth noting, although not yet tested in a SC context, the disruption of the mutp53 interaction with p53/p63/p73; releasing these tumor suppressive proteins from the mutp53 dominant negative effect, using compounds such as 37AA [126], RETRA [127], and LEM2 [128], could also represent an interesting approach to be explored in the near future.

### Crosstalk Between p53 and Vitamin D Receptor in Response to UVR-Induced Skin Damage

In contrast to its potentially harmful effects, UVR also promotes the biosynthesis of vitamin D, which is well-known for its crucial role in calcium homeostasis and anticancer properties by activating the vitamin D receptor (VDR) [129]. VDR acts as a transcriptional regulator, being activated upon high-affinity binding of its naturally occurring ligand 1,25-dihydroxyvitamin D (1,25(OH)2D or calcitriol) at its C-terminal ligand binding domain [130], regulating several genes with protective effects against UVR-induced skin carcinogenesis.

Although the precise mechanism by which vitamin D promotes an efficient DNA repair remains not fully elucidated, accumulated data have demonstrated that vitamin D and related metabolites reduce UVR-induced CPDs [131] and 8-OHdG [132], in several human cells such as keratinocytes [133], melanocytes [134], and fibroblasts [135], the skin of Skh/hr1 mice [136], human skin ex vivo [137], and human volunteers [138]. Consistently, VDR-null mice epidermal explants and cultured keratinocytes from such mice are deficient in the repair of 6-4PP and CPDs by the GG-NER pathway [139]. Additionally, the photoprotective effects of 1,25(OH)2D3 was demonstrated in keratinocytes and UV-irradiated skin mice, with a reduction of CPDs and nitric oxide derivatives, and increased p53 expression [140]. Another study suggested that vitamin D-activated VDR may have a role in the efficient repair of UVR-induced DNA damage by regulating post-translational modifications of the p53 transcriptional target XPC, which is necessary for its proper release from DNA damage sites [139]. In the absence of VDR, XPC retention is associated with the incomplete assembly of the NER pre-incision complex [139].

In addition to promoting DNA repair, vitamin D and derivatives also reduce the production of pro-inflammatory cytokines in human keratinocytes and mouse and human skin [140], delaying the progression of photocarcinogenesis [141]. Similarly to p53, vitamin D and derivatives have been reported to inhibit the NF-κB pathway, downregulating multiple inflammatory cytokines [142]. It was further demonstrated that treatment of UVB-irradiated human epidermal keratinocytes with vitamin D derivatives inhibited inflammatory responses through activation of IκB-α expression and suppression of NF-kB p65 activity and its downstream signaling cytokines, the tumor necrosis factor alpha (TNF-α) and interferon gamma (IFN-γ) [142]. Other studies stated a close association between vitamin D photoprotection and increased p53 nuclear levels, lower apoptotic sunburn cells, and reduced UVB-induced immunosuppression [136].

**Figure 5:**
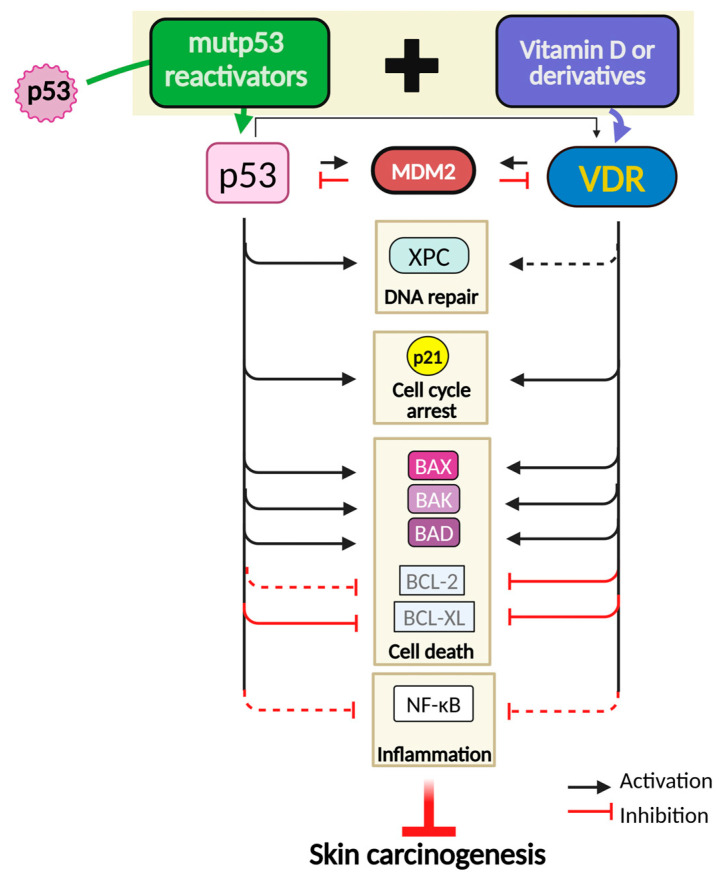
Proposed SC preventive strategy based on p53 and VDR crosstalk, in response to UVR: putative synergic effects between mutp53 reactivators and vitamin D. To foster an efficient prevention against SC, the therapeutic strategy may rely on a synergistic effect combining different mutp53 reactivators in association with vitamin D or derivatives, which will target different UVR-induced cellular processes, inhibiting carcinogenesis. Upon UVR exposure, activated p53 can transactivate the *VDR* gene. Both p53 and VDR promote *MDM2* transcription, engaging in a negative feedback loop with p53 and VDR. Under mild UVR-induced DNA damage, p53 and VDR function together as tumor suppressors. p53 upregulates *XPC* gene expression, while VDR modulates post-translational modifications of XPC, promoting efficient DNA repair. Both p53 and VDR upregulate *CDKN1A* (encoding p21) to induce cell cycle arrest. Under severe DNA damage, p53 and VDR promote the transactivation of genes that stimulate cell death. Both VDR and p53 can inhibit NF-κB, thus exhibiting anti-inflammatory functions. Protein regulators transcriptionally regulated by p53 and VDR are represented by a continuous arrow outlined in a solid box, while those indirectly regulated by p53 or VDR are represented by a dashed arrow (dashed red boxes represent regulators indirectly repressed). BAD, Bcl-2-antagonist of cell death; BAK, Bcl-2 Antagonist/Killer; BAX, Bcl-2-associated X protein; BCL-2 B-cell lymphoma 2; BCL-XL, B-cell lymphoma-extra-large; CDKN*1A*, Cyclin Dependent Kinase Inhibitor 1A; MDM-2, mouse double minute 2; NF-κB, nuclear factor kappa light chain enhancer of activated B; UVR, ultraviolet radiation; VDR, vitamin D receptor; XPC, xeroderma pigmentosum group C protein; TM, ataxia telangiectasia mutated.

Despite the limited data available in SC cells, the tumor suppression network between vitamin D and p53-signaling pathway has been evidenced in skin and other tissues [143] (Figure 5). In fact, it was demonstrated that VDR directly activates the p53-inhibitor *MDM2* through a VDR-response element in the *MDM2* promoter P2 that is near p53 binding sites [144]. This reinforces a potential crosstalk between both transcriptional regulators. Additionally, it was suggested that, like p53, VDR can undergo ubiquitin-dependent degradation by MDM2. As such, VDR and MDM2 may also engage in a negative feedback regulation, at least in tissues in which the *MDM2* gene is transactivated by VDR [143]. VDR also regulates several other genes, in addition to *MDM2*, that are targeted by p53 and its protein family, including the cell cycle regulator *CDKN1A* and several apoptotic effectors [145]. In fact, in a SC context, an association was also demonstrated between VDR and p21, reporting a decreased expression of long non-coding RNA-p21 (*lncRNA-p21*) in VDR-deleted mouse keratinocytes [146]. Additionally, a calcitriol-elicited downregulation of the anti-apoptotic *BCL2* and *BCLXL* and overexpression of the pro-apoptotic *BAX, BAK*, and *BAD* genes have also been described [147]. 1α,25(OH)2 D3-activated apoptosis through caspase effector molecules has also been reported [148]. Importantly, some studies have reported that p63 and p73, and less efficiently p53 itself, can transactivate the *VDR* gene [149]. It has also been shown that mutp53 can disrupt the activity of VDR through various mechanisms, including protein–protein interactions that modify VDR’s localization, stability, or DNA-binding ability. As a result, the transcription of VDR target genes is compromised, impairing the growth-inhibitory effects of vitamin D [150]. Understanding the mechanistic interactions between VDR and mutp53, in the context of NMSC, could provide insights into therapeutic strategies aimed at restoring VDR activity. This restoration could counteract the oncogenic effects of mutp53 and reinstate the tumor-suppressive properties of vitamin D in the skin.

## 4. Conclusions and Future Perspectives

Skin cancer (SC) remains a significant public health concern due to its increasing incidence and potential for substantial morbidity [151]. Collected data have evidenced the key role of mutp53 in skin carcinogenesis processes, particularly of NMSC. Therefore, emerging preventive approaches based on mutp53 reactivators hold significant promise in restoring the native function to mutp53, in the early stages of skin carcinogenesis. By reactivating mutp53, we can potentially delay the need for surgical interventions, offering a novel strategy of SC prevention that may improve the quality and life expectancy of a population increasingly susceptible to UVR-induced SC. Most importantly, an interesting crosstalk between p53 and its VDR transcriptional target is also highlighted in SC suppression, which opens the way to promising pharmacological strategies involving synergistic combinations between mutp53 reactivators and vitamin D.

In conclusion, this review emphasizes the critical role of p53 as a key target in SC prevention, reinforcing the need for further research in this area to prompt the use of p53 chemopreventive agents to counteract the growing number of SC cases.
